# Cavum septum pellucidum and first-episode psychosis: A meta-analysis

**DOI:** 10.1371/journal.pone.0177715

**Published:** 2017-05-17

**Authors:** Hanwen Liu, Ling Li, Li Shen, Xianliang Wang, Yazhu Hou, Zhiqiang Zhao, Lili Gu, Jingyuan Mao

**Affiliations:** 1 Tianjin University of Traditional Chinese Medicine, Tianjin, China; 2 Internal Medicine Unit, First Teaching Hospital of Tianjin University of Traditional Chinese Medicine, Tianjin, China; 3 Chinese Evidence-based Medicine Center, West China Hospital, Sichuan University, Chengdu, Sichuan, China; 4 Department of Psychosomatic Medicine, First Teaching Hospital of Tianjin University of Traditional Chinese Medicine, Tianjin, China; 5 Cardiovascular Department, First Teaching Hospital of Tianjin University of Traditional Chinese Medicine, Tianjin, China; 6 Department of Medical Administration, First Teaching Hospital of Tianjin University of Traditional Chinese Medicine, Tianjin, China; Maastricht University, NETHERLANDS

## Abstract

**Objectives:**

To investigate the prevalence and changes of cavum septum pellucidum (CSP) in first-episode psychosis (FEP) patients.

**Methods:**

Medline, Embase, and the Cochrane Central Register of Controlled Trials (CENTRAL) were searched to identify eligible studies comparing FEP patients and healthy controls from inception to Feb 29, 2016.

**Results:**

Ten cross-sectional studies and three longitudinal studies reported in ten articles met our criteria. Our meta-analysis found no significant differences in the prevalence of either “any CSP” (OR = 1.41; 95% CI 0.90–2.20; p = 0.13; I^2^ = 52.7%) or “large CSP” (OR = 1.10; 95% CI 0.77–1.58; p = 0.59; I^2^ = 24.1%) between FEP patients and healthy controls. However, the heterogeneity analysis of the prevalence of “any CSP” suggested bias in outcome reporting.

**Conclusions:**

The results based on current evidence suggest it is unclear whether “any CSP” is a risk factor for FEP due to the heterogeneity of the studies. There is insufficient evidence to support that “large CSP” is a possible risk factor for FEP.

## Introduction

The cavum septum pellucidum (CSP), commonly used to examine foetal development [1,2], is considered a neurodevelopmental marker later in life [3–6]. A large CSP is often considered indirectly related to psychotic disorders [7–16]. A meta-analysis studying CSP in schizophrenia spectrum disorders (SSD) revealed that normal variations in small-sized CSPs were not related to SSD, whereas a large CSP tended to be a risk factor [17]. In recent years, cross-sectional studies have failed to find significant differences in the prevalence of large CSP between psychosis patients and healthy controls [18–23]. Furthermore, a molecular study reported that a significantly larger CSP was associated with the Disrupted-in-Schizophrenia-1 (DISC1) Ser704Cys polymorphism, although this variant was not found to be unique to schizophrenia patients [18].

The CSP is the space that remains when the leaflets of the septum pellucidum (SP) do not fuse [3]. Serving as a relay station in the limbic system, the SP is thought to connect the hypothalamic autonomic system to the hippocampus, amygdala, and habenula and regulate brain-stem reticular formation [3,24,25]. The SP closes within one month of birth in 15% of subjects and within 6 months in 85% of subjects [26]. The normal fusion of the SP is also associated with an enlargement of the amygdala, hippocampus, and corpus callosum [27]. Recent comparison studies have reported that CSP length in psychosis patients shows negative correlations with the relative volume of the bilateral amygdala, hippocampus, and left posterior parahippocampal gyrus and an association with a shorter adhesio interthalamica (AI) [19,22,28,29]. Others, however, have argued that there is no association between CSP length and the morphology of the anterior cingulate cortex, hippocampus and fornix or the absence of the AI [7,19,20]. Comparisons between first-episode psychosis (FEP) patients and individuals with chronic schizophrenia have suggested that those volumetric reductions may be due to degenerative processes after illness onset [30,31], and chronic schizophrenia patients show an increased prevalence of clinically significant brain abnormalities [32]. Additionally, a meta-analysis of longitudinal magnetic resonance imaging (MRI) studies on patients with schizophrenia and psychotic disorders showed increased rates of lateral ventricle dilation after years of illness [33]. A recent longitudinal study also reported that CSP length increased at a higher rate in FEP patients, which may explain the higher prevalence of CSP in chronic cases [34], whereas increased CSP length in patients may be caused by the effects of antipsychotics or the duration of illness [35].

Thus, whether the CSP may serve as a risk factor for psychosis or is only a reflection of neuroanatomical changes in individuals with chronic psychotic disorders remains ambiguous. Therefore, we conducted a meta-analysis to assess the association between the CSP and FEP.

## Methods

We conducted this study according to the standards of the Meta-analysis of Observational Studies in Epidemiology (MOOSE) guidelines [36]. We searched records from Medline, Embase, and the Cochrane Central Register of Controlled Trials (CENTRAL) from inception to Feb 29, 2016 using the following terms: “septum pellucidum”, “septi pellucidi”, “psychosis”, “psychotic”, and “schizophrenia” (see search strategies in the appendix). The inclusion criteria were as follows: 1) use of MRI to assess the CSP; 2) a population diagnosed with FEP; 3) a comparative group of healthy subjects; and 4) publication in English. The exclusion criteria were as follows: studies on infants or children. Teams of two trained and paired reviewers screened eligible titles, abstracts, and full texts independently, evaluated the risk of bias, and collated data from each study meeting our criteria. Disagreements between reviewers were resolved through discussion or judged by a third reviewer. Stata version 12.0 was used to analyse the outcome data. Dichotomous data were pooled using the odds ratios (ORs), and the continuous data were pooled using the mean differences (MDs) and associated 95% confidence intervals (CIs). The heterogeneity of the statistical models was examined via the χ^2^ test and the I^2^ statistic. The random effects model was used when I^2^ >50%, and the fixed effects model was used when I^2^<50% [37]. A funnel plot and Egger’s test were used to examine publication bias. To explore the source of heterogeneity, subgroup analyses for the different CSP measurement and assessment (qualitative or quantitative) methods were conducted. A meta-regression model was estimated using the residual maximum likelihood (REML) method, with the different assessment methods, CSP prevalence in the healthy controls and key sample characteristics as predictors. A sensitivity analysis was conducted using the leave-one-out method.

## Results

**Fig 1:**
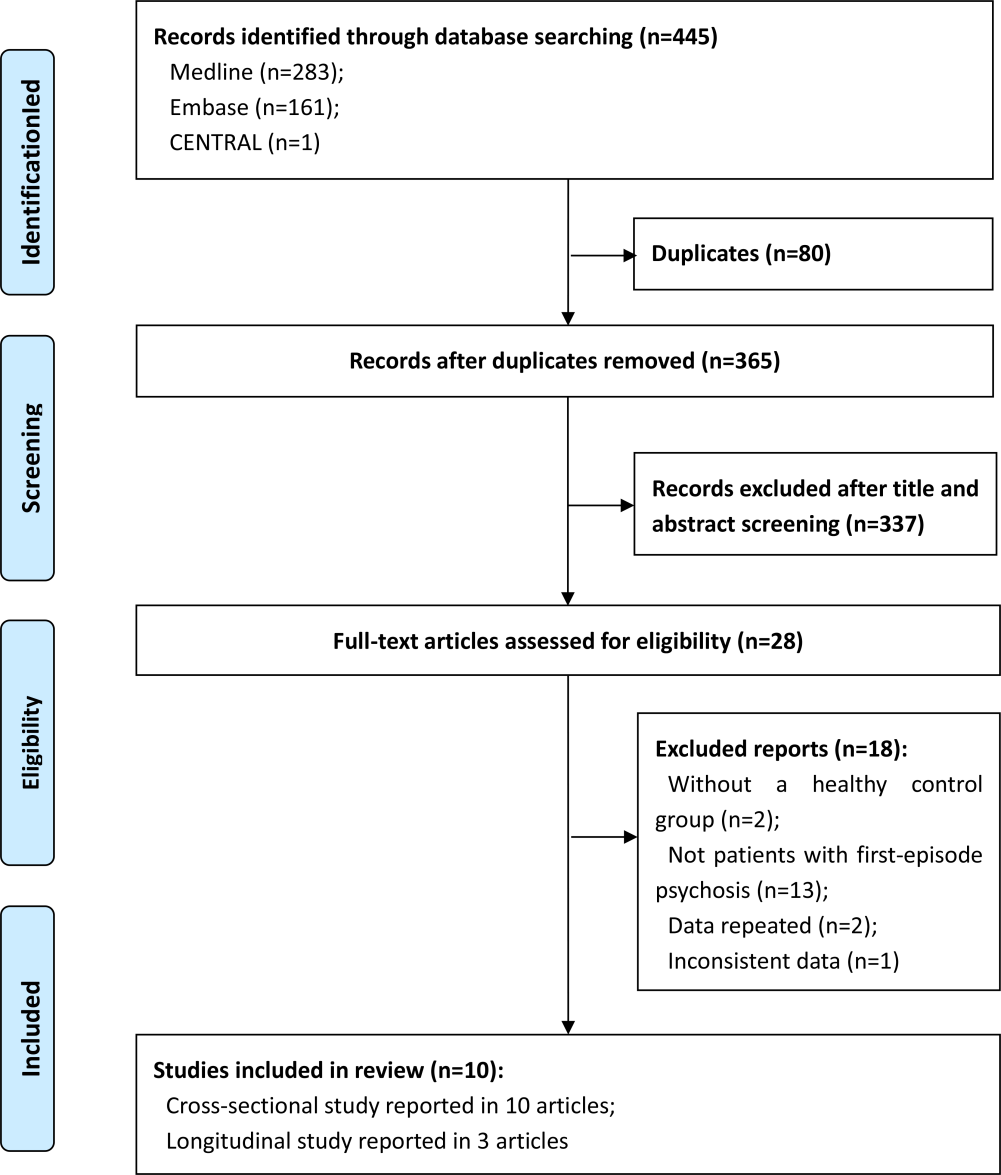
Article selection flow diagram.

A total of 445 articles were identified through database searches. Of these, 80 duplicates were removed, 337 records were excluded after the title and abstract screening, and 28 potentially eligible records remained. After the full text screening, we excluded 18 articles for the following reasons: the lack of a group of healthy controls [12,38]; did not contain FEP subjects [7,10,11,13,18,22,23,39–44]; sample was used in another study [9,15]; or inconsistent data [21]. Finally, we included 10 articles, three of which reported follow-up longitudinal studies assessing CSP length [19,29,34]. We also used the baseline data from 10 cross-sectional studies [8,14,16,19,20,27–29,34,45] to compare the prevalence of CSP in FEP patients with that in healthy controls (Fig 1).

### Evidence from cross-sectional studies

The 10 cross-sectional studies enrolled 749 FEP patients (mean age range, 21.5–30.3 years; mean range of FEP duration, 0.1–1.0 years; Table 1) and 727 healthy controls. To assess CSPs, four studies (thickness of MRI slices >3 mm) used qualitative assessments, including three studies that used grading systems based on visual inspection of coronal MRI slices [14,16,27] and one that used the “with/without” method [45]. The other six studies used quantitative assessment, by multiplying the number of coronal MRI slices (thickness ≤1.5 mm) on which the CSP could be visualized [8,20,29,34] or measuring the anteroposterior length of the CSP in millimetres [19,28] (Table 2). Three of the 10 studies reported a significantly higher prevalence of “any CSP” in FEP patients [14,16,28], and one study reported a significantly higher prevalence of “large CSP” in FEP patients [8]. The other six studies found no significant difference in the prevalence of either “any CSP” or “large CSP” between FEP patients and healthy controls [19,20,27,29,34,45] (Table 2).

**Table 1:** Sample characteristics of the cross-sectional studies.

Reference (year)	Country	FEP Patients						Healthy controls		
		Type	Diagnostic criteria	N (M/F)	Age, mean ± SD (years)	FEP duration, mean ± SD (years)	Medication	N (M/F)	Age, mean ± SD (years)	Origin
Degreef (1992) [16]	USA	FESZ	RDC	62 (33/29)	24.1 ± 5.8	1.0±1.7	Drug-naïve	46 (22/24)	28.8 ± 7.5	Hospital staff; community
DeLisi (1993) [14]	USA	FEP	DSM-III-R	85 (48/37)	26.6 ± 7.3	NR	NR	47 (29/18)	26.6 ± 6.6	Community
Keshavan (2002) [27]	USA	FESZ & FESZA	DSM-IV	40 (25/15)	24.7 ± 7.5	NR	Drug-naïve	59 (29/30)	21.4 ± 7.5	Community
Kasai (2004) [8]	USA	FESZ	DSM-IV	33 (28/5)	24.7 ± 6.5	NR	Yes	56 (44/12)	24.0 ± 3.9	Community
		FEAFP	DSM-IV	41 (31/10)	22.8 ± 4.6	NR	Yes			
Borgwardt (2006) [45]	Switzerland	FEP	ICD-10	30 (22/8)	30.3 ± 6.9	NR	Yes	26 (17/9)	22.5 ± 4.4	School students, hospital staff, community
Takahashi (2008) [20]	Australia	FEP	DSM-III-R	162 (108/54)	21.5 ± 3.4	0.1 ± 0.2	Yes	87 (55/32)	26.9 ± 10.1	Hospital staff, community
Davidson (2012) [19]	USA	FESZ	DSM-IV	25 (21/4)	25.9 ± 8.5	0.3 ± 0.3 ^a^	Yes	25 (21/4)	26.2 ± 3.5	Community
Trzesniak (2012) [34]	Brazil	FEP	DSM-IV	122 (66/56)	28.6 ± 8.4	0.5 (0.6) ^b^	Yes	94 (53/41)	30.2 ± 8.4	Community
Takahashi (2013) [29]	Japan	FESZ	ICD-10	64 (37/27)	24.0 ± 4.7	0.9 ± 1.0	Yes	64 (37/27)	25.1 ± 5.0	Community, hospital staff, university students
Landin-Romero (2016) [28]	Spain	FEP	DSM-IV	85 (58/27)	26.9 ± 8.2	0.5 ± 1.3	NR	223 (99/124)	36.0 ± 11.3	Hospital staff, community

Abbreviations: CTRL, controls; FEAFP, first-episode affective psychosis; FEP, first-episode psychosis; FESZ, first-episode schizophrenia; FESZA, first-episode schizoaffective disorders; NR, not reported; RDC, Research Diagnostic Criteria.

^a^ 13 of 25 participants available

^b^ Median (IQR) of FEP duration.

**Table 2:** CSP prevalence and results of the cross-sectional studies.

Reference (year)	MRI Tesla/ slice thickness	Measurement of CSP	Large CSP criteria	FEP Patients					Healthy controls				Results/findings
				Type	N (M/F)	Any CSP (%) ^b^	Large CSP (%) ^d^	CSP length, mean ± SD (mm)	N (M/F)	Any CSP (%) ^b^	Large CSP (%) ^d^	CSP length, mean ± SD (mm)	
Degreef (1992) [16]	1.0 T/3.1 mm	QLA (visual inspection Grade 0–3)	Grade 3	FESZ	62 (33/29)	35.5	3.2	NR	46 (22/24)	15.2	0.0	NR	Any CSP: FESZ↑; large CSP: NS
DeLisi (1993) [14]	1.5 T/5.0 mm (2.0 mm ^a^)	QLA (visual inspection Grade 0–3)	Grade 3	FEP	85 (48/37)	44.7	1.2	NR	47 (29/18)	29.8	0.0	NR	Any CSP: FEP↑; large CSP: NS
Keshavan (2002) [27]	1.5 T/3.0 mm	QLA (visual inspection Grade 1–3)	Grade 3	FESZ & FESZA	40 (25/15)	10.0	2.5	NR	59 (29/30)	11.9	0.0	NR	Any CSP: NS; large CSP: NS
Kasai (2004) [8]	1.5 T/0.9375 mm	QNA (number of 0.9375-mm slices)	≥N slices (5.6 mm)	FESZ	33 (28/5)	69.7	18.2	NR	56 (44/12)	87.5	7.1	NR	Any CSP: NS; large CSP:↑
				FEAFP	41 (31/10)	80.5	14.6	NR					
Borgwardt (2006) [45]	1.5 T/3.0 mm	QLA (visual inspection with/without)	Above normal	FEP	30 (22/8)	3.3	0.0	NR	26 (17/9)	0.0	0.0	NR	Any CSP: NS; large CSP: NS
Takahashi (2008) [20]	1.5 T/0.9375 mm	QNA (number of 0.9375-mm slices)	≥N slices (5.6 mm)	FEP	162 (108/54)	89.5	9.3	NR	87 (55/32)	89.7	11.5	NR	Any CSP: NS; large CSP: NS
Davidson (2012) [19]	1.5 T/1.5 mm	QNA (measured in millimetres)	≥N mm	FESZ	25 (21/4)	64.0	0.0	1.44 ± 1.33	25 (21/4)	76.0	12.0	3.12 ± 3.11	Any CSP: NS; large CSP: NS
Trzesniak (2012) [34]	1.5 T/1.5 mm	QNA (number of 1.5-mm slices)	≥N slices (6 mm)	FEP	122 (66/56)	94.3	30.3	4.44 ± 1.93 ^e^^;^ ^f^	94 (53/41)	88.3	29.8	4.62 ± 1.95 ^e^^;^ ^g^	Any CSP: NS; large CSP: NS
Takahashi (2013) [29]	1.5 T/1.0 mm	QNA (number of 1.0-mm slices)	≥N slices (6 mm)	FESZ	64 (37/27)	87.5 ^c^	4.7	3.10 ± 6.50	64 (37/27)	84.4 ^c^	12.5	4.70 ± 10.10	Any CSP: NS; large CSP: NS
Landin-Romero (2016) [28]	1.5 T/1.0 mm	QNA (measured in millimetres)	>5 mm	FEP	85 (58/27)	56.5	11.8 ^c^	NR	223 (99/124)	31.8	6.3	NR	Any CSP: FEP↑; large CSP: NR

Abbreviations: CTRL, controls; CSP, cavum septum pellucidum; FEAFP, first-episode affective psychosis; FEP, first-episode psychosis; FESZ, first-episode schizophrenia; FESZA, first-episode schizoaffective disorders; NR, not reported; NS, not significance; QLA, qualitative assessment; QNA, quantitative assessment; RDC, Research Diagnostic Criteria.

^a^ Selection gap

^b^ calculated by: 100 x (number of subjects with CSP/number of all subjects)

^c^ original data

^d^ calculated by: 100 x (number of subjects with large CSP/number of all subjects)

^e^ transformed from (ln) mean ± SD

^f^ 112 of 122 subjects available

^g^ 80 of 94 controls available.

#### Prevalence of “any CSP” in FEP Patients

**Fig 2:**
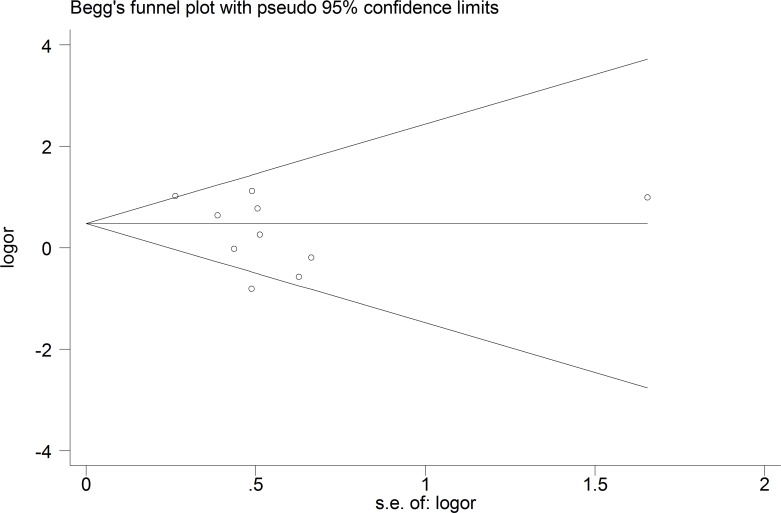
Funnel plot of the prevalence of “any CSP” in the FEP patients.

**Fig 3:**
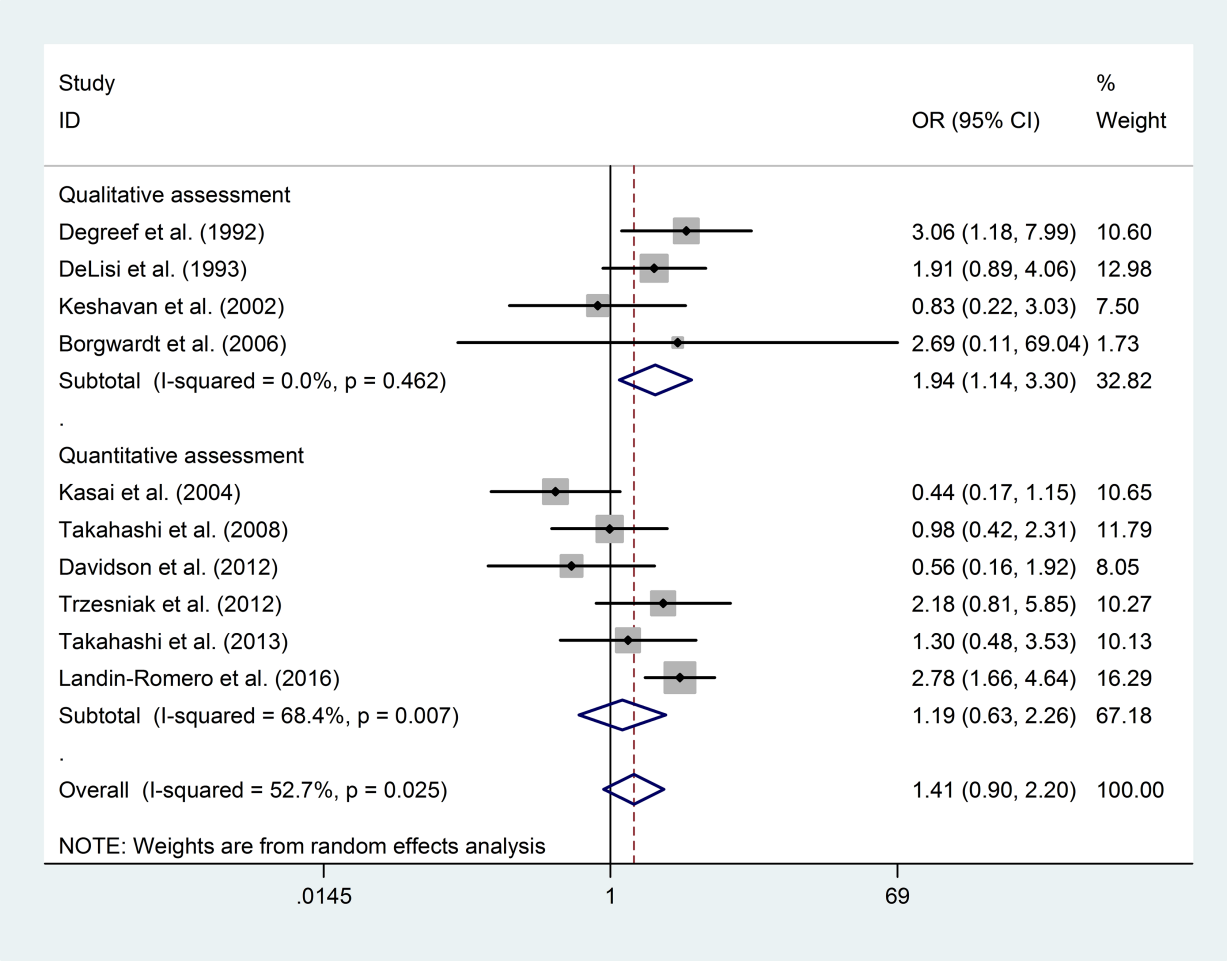
Prevalence of “any CSP” in FEP patients and healthy controls.

The funnel plot and publication bias test for the prevalence of “any CSP” showed no significant results (Fig 2, Egger's test p = 0.20). The group of tests that included the qualitative assessment showed a significantly higher prevalence of “any CSP” in the FEP patients than in the healthy controls (OR = 1.94; 95% CI 1.14–3.30; p = 0.02; I^2^ = 0%), whereas the group of tests that included the quantitative assessment showed no significant results (OR = 1.19; 95% CI 0.63–2.26; p = 0.59; I^2^ = 68.4%). The overall risk of “any CSP” in the FEP patients was not significantly different from that in the healthy controls (OR = 1.41; 95% CI 0.90–2.20; p = 0.13; I^2^ = 52.7%) (Fig 3).

**Fig 4:**
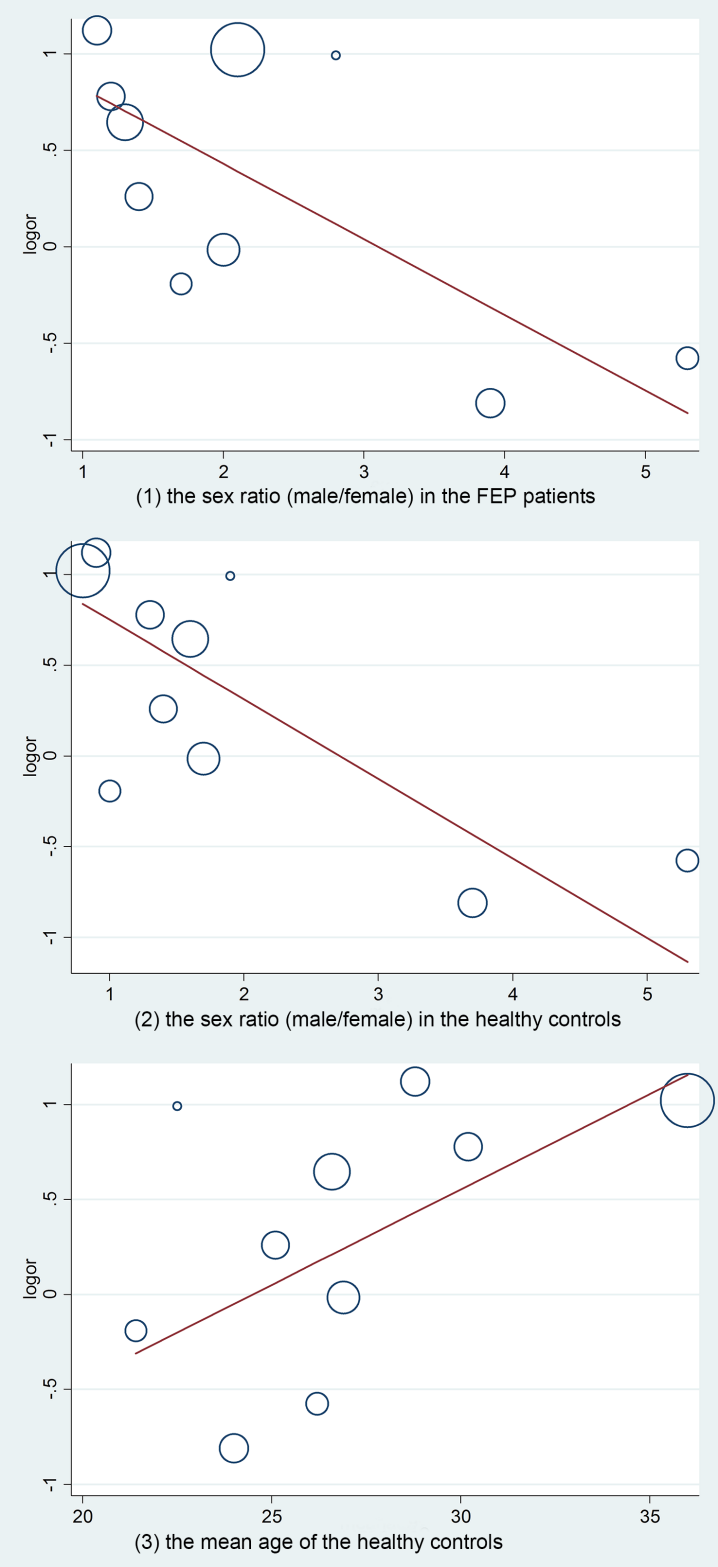
Meta-regression of “any CSP” according to (1) the sex ratio (male/female) in the FEP patients; (2) the sex ratio (male/female) in the healthy controls; and (3) the mean age of the healthy controls.

The univariable meta-regression analyses showed no statistically significant association between the OR of “any CSP” and the four predictors (assessment method: p = 0.42; publication year: p = 0.80; prevalence of CSP in the healthy controls: p = 0.08; mean age of the FEP patients: p = 0.15), whereas the other three predictors (sex ratio of the FEP patients: p = 0.04; sex ratio of the healthy controls: p = 0.01; mean age of the healthy controls: p = 0.02) showed statistically significant associations with the OR of “any CSP”. Some of the studies reported an extreme gender imbalance (male/female>2) in the FEP patients [8,19,20,28,45] (Fig 4 (1)) and in the healthy controls [8,14,16,19,20,27–29,34,45] (Fig 4 (2)). One study [28] reported a much older mean age of the healthy controls than the other studies (Fig 4(3)) and of the FEP patients in the same study (Table 1).

The sensitivity analysis conducted using the leave-one-out method suggested instability in outcome reporting. The prevalence of “any CSP” was higher in the FEP patients (OR = 1.82; 95% CI 1.36–2.43; p<0.001; I^2^ = 29.3%) after one study [8] was excluded.

#### Prevalence of “large CSP” in the FEP Patients

**Fig 5:**
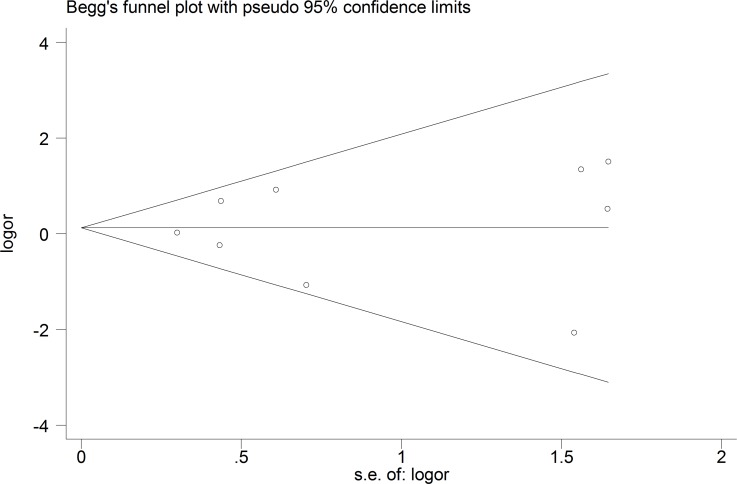
Funnel plot of the prevalence of “large CSP” in the FEP patients.

**Fig 6:**
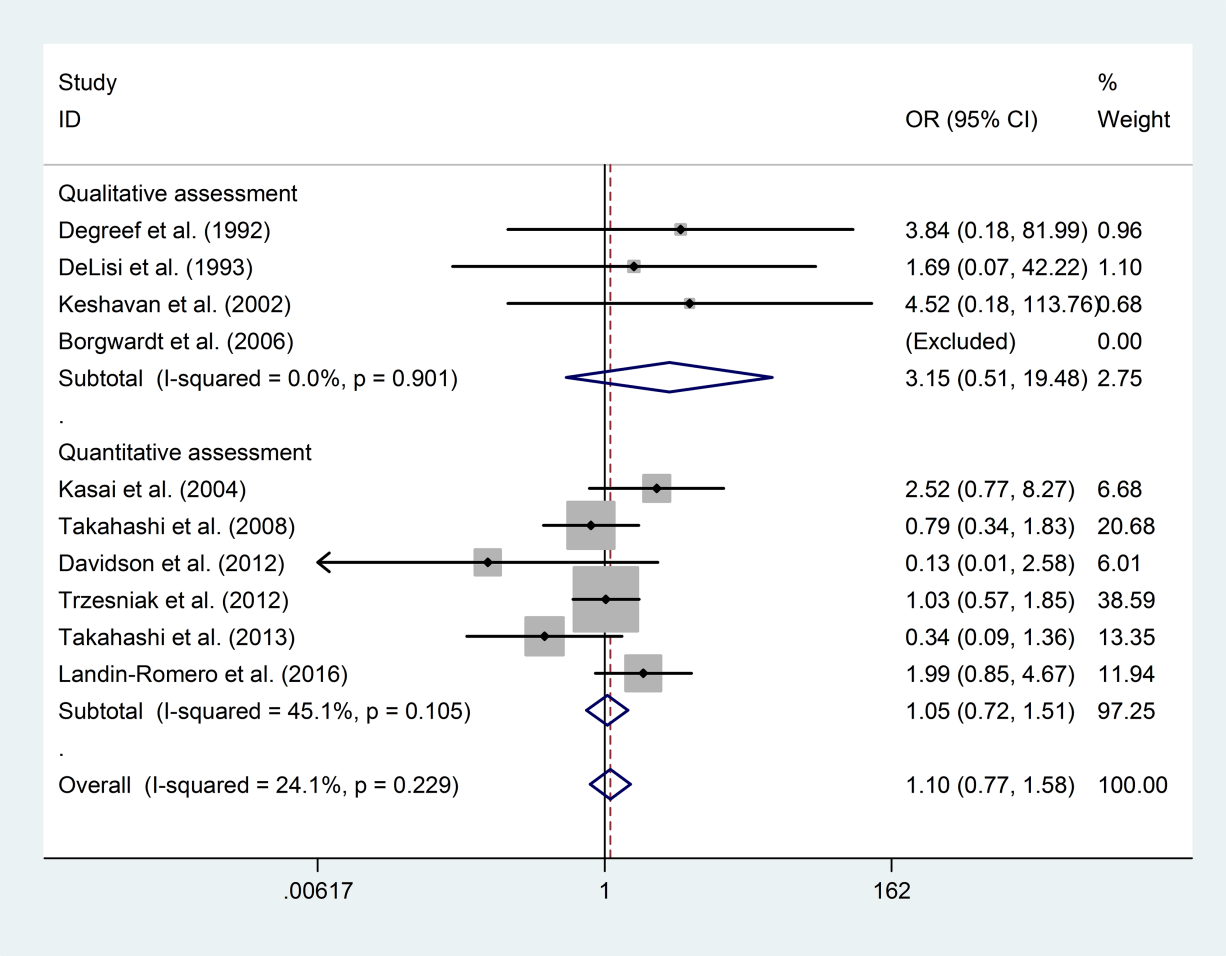
Prevalence of “large CSP” in the FEP patients and healthy controls.

When comparing the prevalence of “large CSP” between the FEP patients and healthy controls, the funnel plot and publication bias test showed no significant results (Fig 5, Egger's test p = 0.91). In the subgroup analyses of the risk of “large CSP”, neither the qualitative assessment group (OR = 3.15; 95% CI 0.51–19.48; p = 0.22; I^2^ = 0%) nor the quantitative assessment group (OR = 1.05; 95% CI 0.72–1.51; p = 0.81; I^2^ = 45.1%) showed significant differences, and there was no significant difference in the overall risk (OR = 1.10; 95% CI 0.77–1.58; p = 0.59; I^2^ = 24.1%) (Fig 6).

The univariable meta-regression analyses showed no statistically significant association between the OR of “large CSP” and the seven predictors (assessment method: p = 0.32; publication year: p = 0.37; prevalence of CSP in the healthy controls: p = 0.46; sex ratio in the FEP patients: p = 0.80; sex ratio in the healthy controls: p = 0.68; mean age of the FEP patients: p = 0.75; and mean age of the healthy controls: p = 0.50). The sensitivity analysis conducted using the leave-one-out method suggested no significant differences in outcome between studies.

#### Length of the CSP in FEP Patients

**Fig 7:**
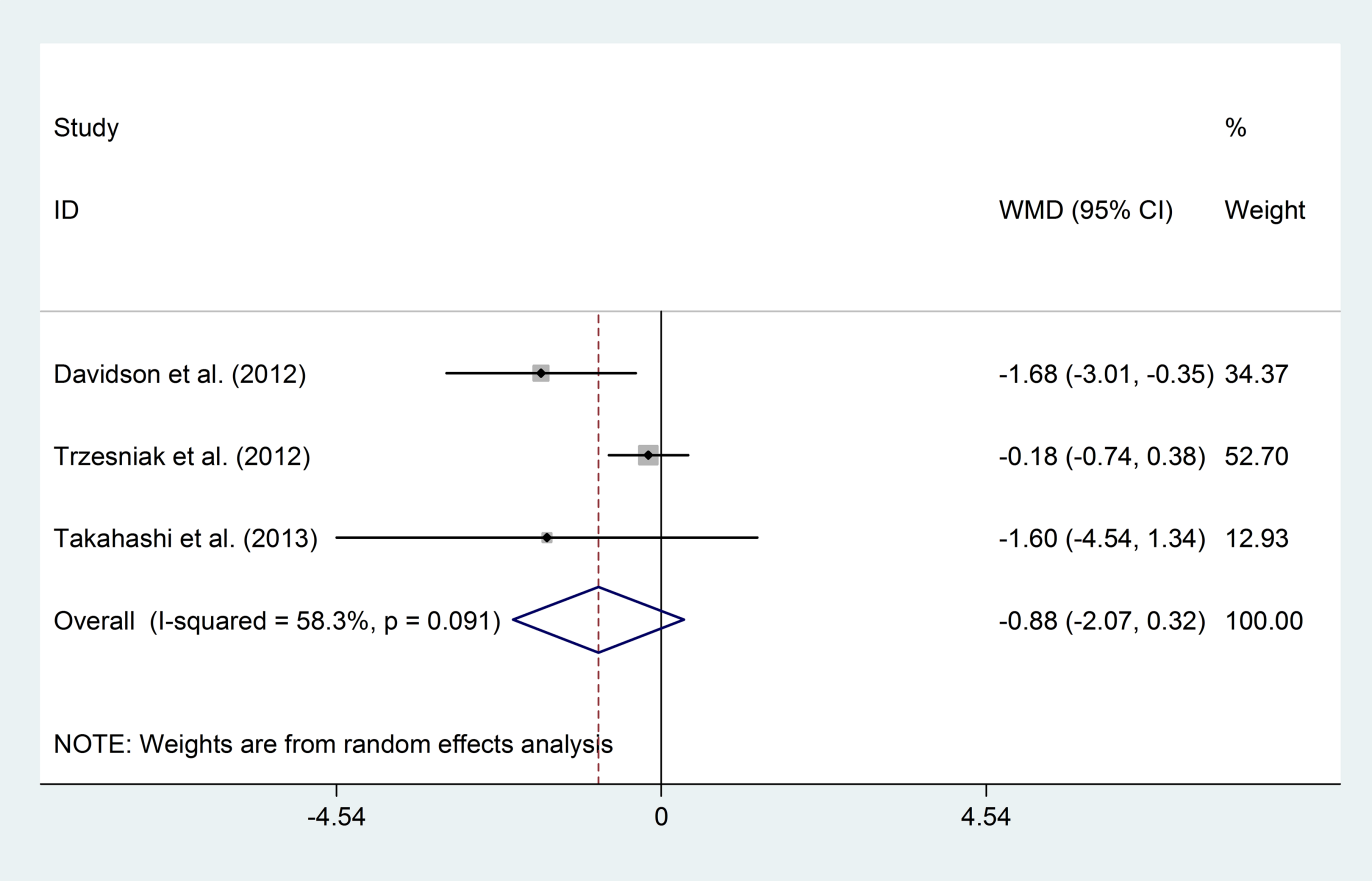
CSP length in the FEP patients and healthy controls.

Three studies [19,29,34] reported eligible data for comparing the length of the CSP in the FEP patients and healthy controls, and they showed no significant difference (mean difference = -0.88; 95% CI -2.07–0.32; p = 0.15; I^2^ = 58.3%) (Fig 7).

#### Prevalence of “Any” CSP” and “Large CSP” in First-Episode Schizophrenia (FESZ) Patients

**Fig 8:**
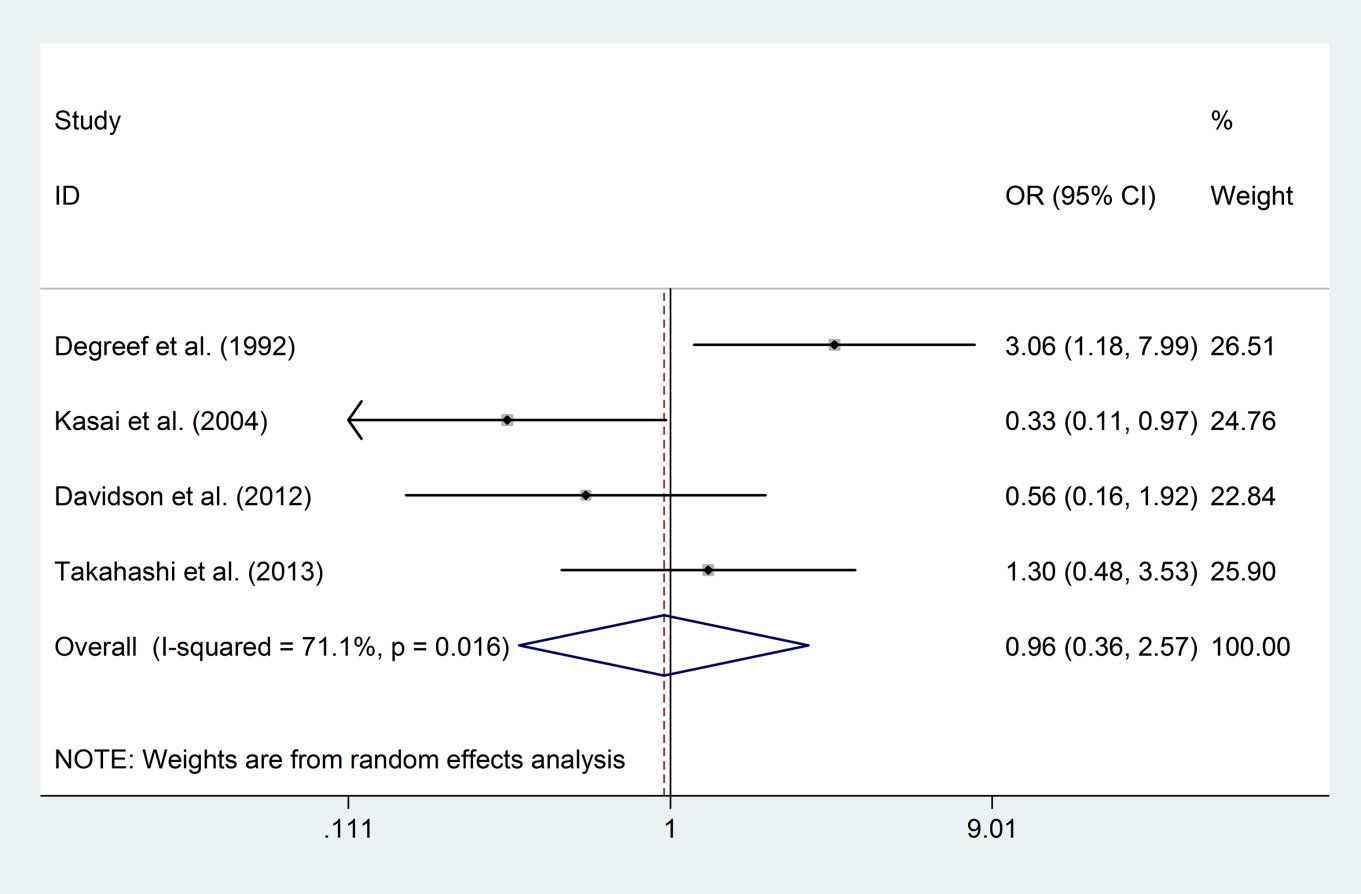
Prevalence of “any CSP” in FESZ patients and healthy controls.

**Fig 9:**
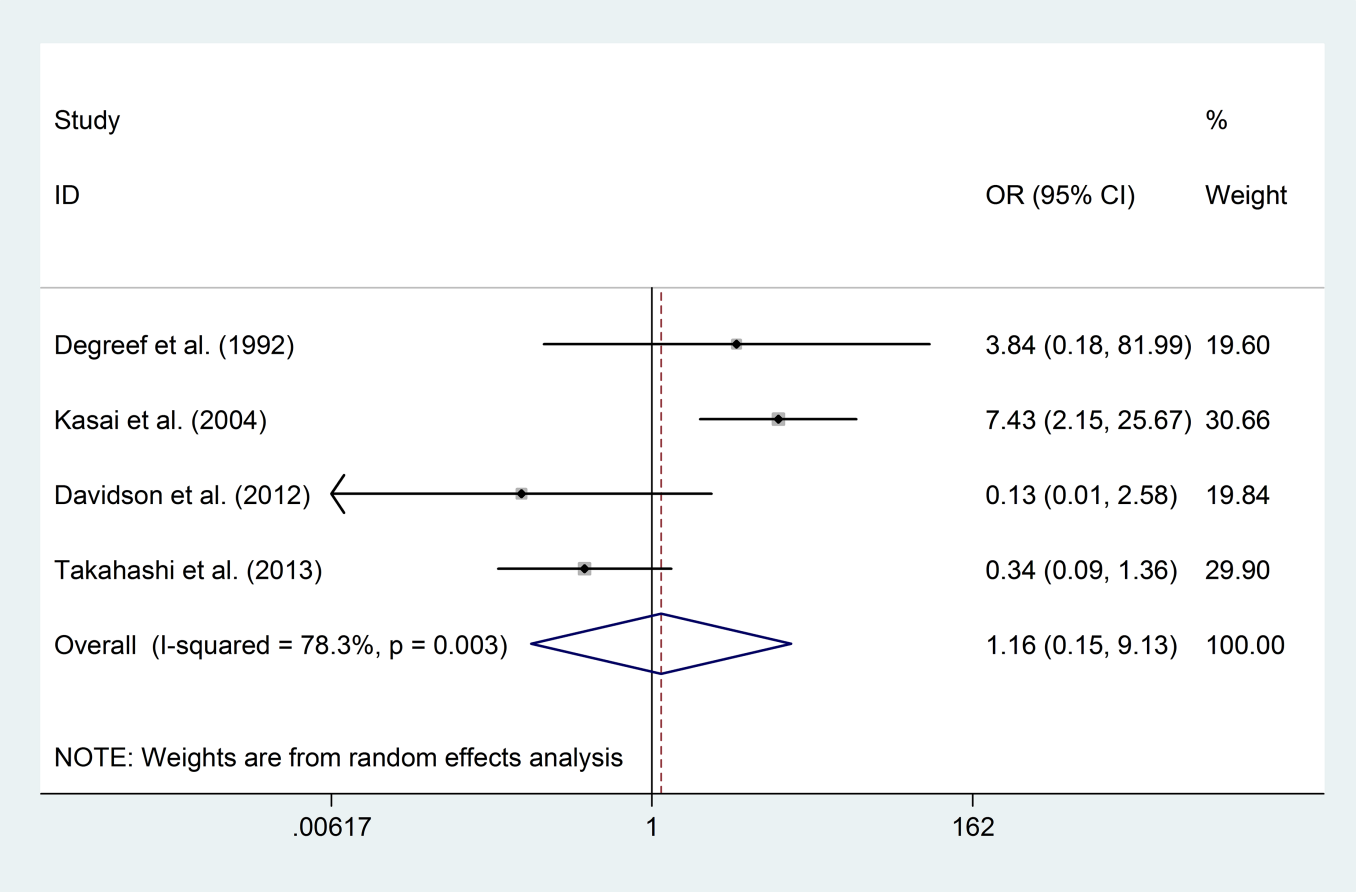
Prevalence of “large CSP” in FESZ patients and healthy controls.

Although six studies [8,16,19,20,29,34] reported 292 first-episode schizophrenia (FESZ) patients in total, four studies [8,16,19,29], which included 184 FESZ patients and 191 healthy controls, had eligible data for comparing the prevalence of “any CSP” and “large CSP”. The risks of “any CSP” (OR = 0.96; 95% CI 0.36–2.57; p = 0.93; I^2^ = 71.1%) and “large CSP” (OR = 1.16; 95% CI 0.15–9.13; p = 0.89; I^2^ = 78.3%) between the FESZ patients and healthy controls showed no significant differences (Fig 8; Fig 9).

### Evidence from longitudinal studies

Three studies included eligible baseline and follow-up reports [19,29,34], two studies maintained the same sample subjects [19,29], and one study was able to assess some of the subjects after follow-up [34]. Therefore, to study changes in CSP length after follow-up (mean duration range 1.1–2.6 years), a total of 120 patients were assessed (Table 3). Two studies reported that the CSP length of subjects was stable in both FEP patients and healthy controls [19,29]. With a larger sample, another study [34] reported that CSP length increased in both FEP patients and healthy controls but increased more significantly in FEP patients (Table 4).

**Table 3:** Sample characteristics of longitudinal studies.

Reference (year)	Country	FEP Patients						Healthy controls		
		Type	Diagnosis criteria	N (M/F) (Baseline/ Follow-up)	Age (Baseline), mean ± SD (years)	FEP duration, (Baseline) mean ± SD (years)	Medication	N (M/F) (Baseline/ Follow-up)	Age (Baseline), mean ± SD (years)	Origin
Davidson (2012) [19]	USA	FESZ	DSM-IV	25 (21/4)/25 (21/4)	25.9 ± 8.5	0.3 ± 0.3 ^b^	Yes	25 (21/4)/25 (21/4)	26.2 ± 3.5	Community
Trzesniak (2012) [34]	Brazil	FEP	DSM-IV	112/75 ^a^	28.6 ± 8.4	0.5 (0.6) ^c^	Yes	80/45 ^a^	30.2 ± 8.4	Community
Takahashi (2013) [29]	Japan	FESZ	ICD-10	20 (14/6)/20 (14/6)	23.8 ± 5.0	0.85 ± 0.78	Yes	21 (13/8)/21 (13/8)	24.5 ± 5.0	Community, hospital staff, university students

Abbreviations: CTRL, controls; FEP, first-episode psychosis; FESZ, first-episode schizophrenia.

^a^ Follow-up assessment subjects

^b^ 13 of 25 subjects available

^c^ median (IQR) of FEP duration.

**Table 4:** CSP length and results of longitudinal studies.

Reference (year)	MRI Tesla/ slice thickness	Follow-up mean ± SD (years)	FEP Patients					Healthy controls				Results/findings
			Type	N (M/F) Baseline/ Follow-up	CSP length (Baseline), mean ± SD (mm)	CSP length (Follow-up), mean ± SD (mm)	Mean differences ^e^ ± SD of differences ^f^ (mm)	N (M/F) Baseline/ Follow-up	CSP length (Baseline), mean ± SD (mm)	CSP length (Follow-up), mean ± SD (mm)	Mean differences ^e^ ± SD of differences ^f^ (mm)	
Davidson (2012) [19]	1.5 T/1.5 mm	1.6 ± 1.4	FESZ	25 (21/4)/ 25 (21/4)	1.44 ± 1.33	1.56 ± 1.53	0.12 ± 1.44	25 (21/4)/ 25 (21/4)	3.12 ± 3.11	3.28 ± 3.27	0.16 ± 3.19	CSP length: both NS
Trzesniak (2012) [34]	1.5 T/1.5 mm	1.1 (0.3) ^a^	FEP	112/75 ^c^	4.44 ± 1.93 ^d^	5.93 ± 1.70 ^d^	1.49 ± 1.83	80/45 ^a^	4.62 ± 1.95 ^d^	5.70 ± 2.01 ^d^	1.08 ± 1.98	CSP length: both↑(FEP > CTRL)
Takahashi (2013) [29]	1.5 T/1.0 mm	2.6 ± 0.6 ^b^	FESZ	20 (14/6)/ 20 (14/6)	3.40 ± 5.90	3.20 ± 5.70	-0.20 ± 5.80	21 (13/8)/ 21 (13/8)	4.80 ± 11.40	4.90 ± 11.60	0.10 ± 11.50	CSP length: both NS

Abbreviations: CTRL, controls; CSP, cavum septum pellucidum; FEP, first-episode psychosis; FESZ, first-episode schizophrenia; NS, not significance.

^a^ median (IQR) of follow-up years

^b^ combine FESZ and CTRL

^c^ Follow-up assessment subjects

^d^ transformed from (ln) mean ± SD

^e^ mean differences = mean (follow-up)—mean (baseline)

^f^ a correlation coefficient of 0.5 was imputed.

#### Increases in CSP length in FEP Patients

**Fig 10:**
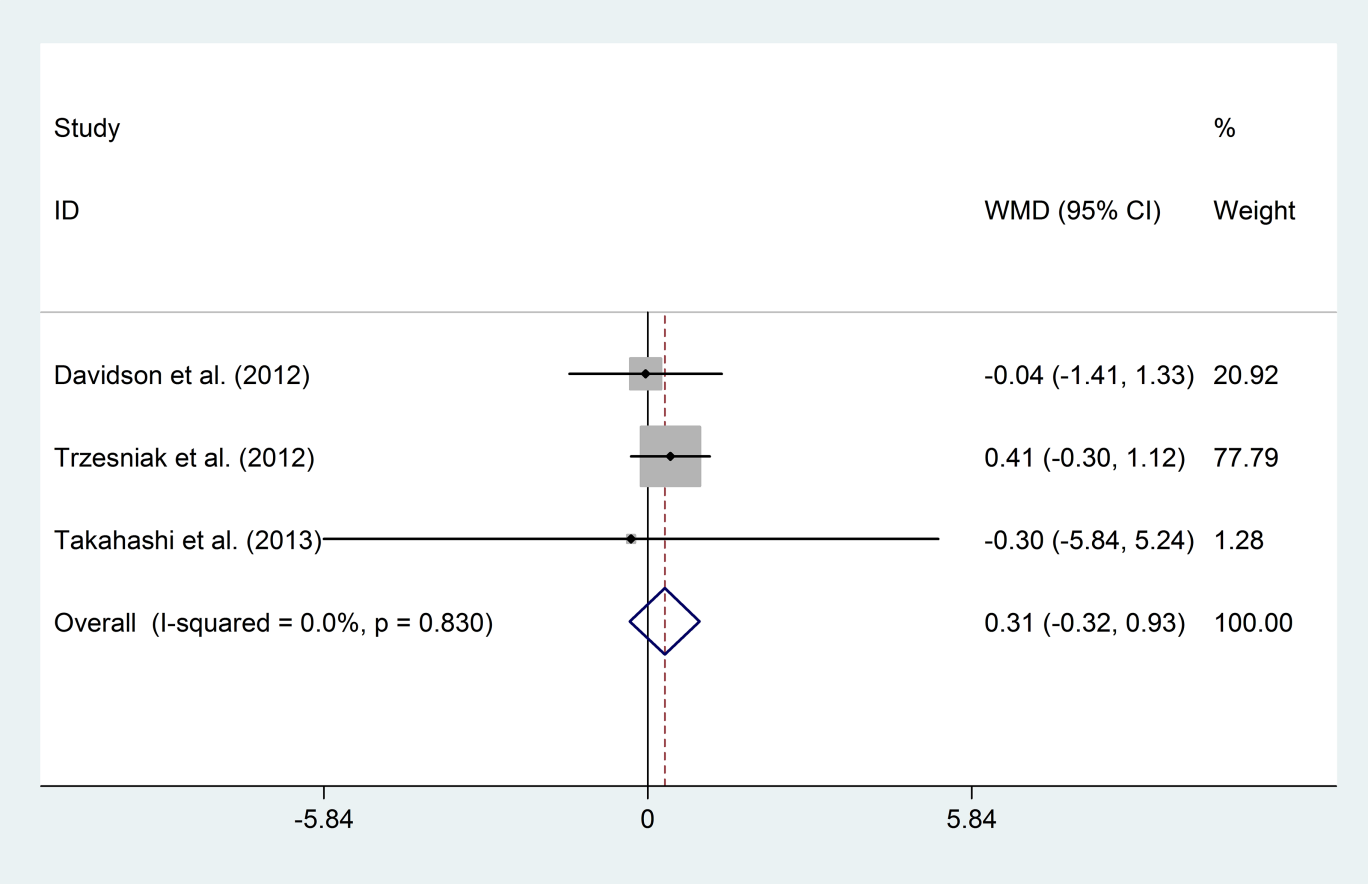
Increases in the CSP length in the FEP patients and healthy controls.

Data on increases in CSP length after follow-up were transformed into the mean difference ± SD of the differences in each group (Table 4). A comparison of increases in CSP length between FEP patients and healthy controls showed no significant differences (mean difference = 0.31, 95% CI -0.32–0.93, p = 0.34, I^2^ = 0%) (Fig 10).

## Discussion

### Main findings

The results from recent cross-sectional studies [8,14,16,19,20,27–29,34,45] showed that the CSP prevalence and length were not statistically different between FEP patients and healthy controls. However, the a meta-regression analysis comparing the prevalence of “any CSP” between FEP patients and healthy controls according to the sex ratio of both groups and the mean age of the healthy controls suggested study heterogeneity, and the sensitivity analysis suggested that the OR was not stable. Our study found no evidence in support of a significant difference in the prevalence of “large CSP” between FEP patients and healthy controls. Meanwhile, the prevalence of CSP was not higher in the FESZ group. After a few years of follow-up, the evidence from recent longitudinal studies [19,29,34] showed no significant difference in the magnitude of the increase in CSP length between FEP patients and healthy controls.

### Study of FEP patients

Our study included only FEP patients for several reasons. First, some studies suggested that antipsychotics were associated with smaller brain volumes and larger temporal cerebrospinal fluid (CSF) volumes [46,47]. Second, even without antipsychotic intervention, patients with long-term untreated psychoses also exhibit subtle morphometric changes in their brains [48]. In the 10 included studies [8,14,16,19,20,27–29,34,45], the FEP patients (mean age range, 21.5–30.3 years; Table 1) were relatively younger when compared to chronic schizophrenia patients (mean age range, 28.0–52.9 years) in the CSP studies [7,9,10,13,16,19,20,22,23,28,40,41,43,44]. The mean FEP duration ranged from 0.1–1.0 years in the cross-sectional studies (Table 1) and from 0.3–0.85 years for the baseline of longitudinal studies (Table 3). Additionally, two studies reported on antipsychotic-naïve patients [16,27], and six studies [8,19,20,29,34,45] reported on short-term antipsychotic treatments before MRI scanning in several cases. Theoretically, the inclusion of only FEP patients decreased the confounding effects of illness duration and medication use. Thus, it would be an advantage in exploring whether a CSP presents as a possible neurodevelopmental marker or an outcome of illness progression.

### Measurement and assessment of the CSP

One significant limitation in our analysis was the mixture of MRI measurements, which may result a variations in CSP prevalence because of potentially missing smaller CSPs [20,21]. In the cross-sectional studies, the prevalence of “any CSP” in FEP patients varied from 3.3% [45] to 94.3% [34] and from 0.0% [45] to 89.7% [20] in healthy controls. However, earlier studies [14,16,27,45] used thicker (≥3.0 mm) MRI slices and reported a lower prevalence of “any CSP” than later studies (MRI thickness approximately 1.0 mm).

Different assessments of CSP were combined with the MRI methods. Earlier studies used qualitative assessments. For example, Degreef [16] used visual inspection with a grading system from 0 to 3 (representing absent, questionable, small, moderate and large), and other studies [14,27] used similar methods, except for that of Borgwardt [45], which detected a normal CSP variant in only one FEP patient using the with/without classification in a qualitative approach. However, both the thickness of MRI slices and the measurements seemed unsatisfactory for identifying CSP prevalence. Moreover, visual inspections were actually based on the width of the CSP rather than the length measurement in the studies using quantitative assessments.

As described by Nopoulos [10], the length of the CSP can be calculated based on the number of thin-slice MRI slices, and later studies preferred this quantitative method. However, this method remains questionable. Davidson [19] claimed that a normal CSP may be associated with the SP due to its lengthwise stretching, and the assessment of only CSP length may not be sensitive enough to detect existing changes [29]. For example, Choi [49] conducted a grading measurement of length, width and overall size, and de Souza Crippa [7] measured the volume of the CSP using voxels.

To explore the source of heterogeneity of the findings, we performed subgroup analyses of the outcomes reported according to the different CSP measurement and assessment (qualitative vs. quantitative) methods. Our analysis of “any CSP” prevalence (Fig 3) showed that although the overall comparison between FEP patients and healthy controls showed no significant difference, the subgroup using qualitative assessments did show a significant difference in FEP patients. However, this difference has minimal practical value because of the small sample size. Furthermore, the study heterogeneity estimated by the sensitivity analysis may also be explained by the measurement and assessment methods. After the exclusion of one study [8] (MRI thickness = 0.9375 mm) that detected a higher prevalence of normal variance of CSP, the prevalence of “any CSP” was significantly higher in FEP patients than in healthy controls.

### Other limitations

The instability of diagnoses could be another limitation in this analysis. A meta-analysis estimating the diagnostic stability of FEP reported that the prospective diagnostic stability in schizophrenia was high, with no significant ICD/DSM difference, while the stabilities of other first-episode psychotic diagnoses were low [50]. From the ten included studies, 292 FEP patients were diagnosed with FESZ [8,16,19,20,29,34], and the other subjects were diagnosed with a mixture of first-episode schizophrenia spectrum disorders, affective psychosis, or psychoses not otherwise specified [8,14,20,27,28,34,45]; seven studies reported a confirmation of diagnoses after follow-up [8,16,19,20,27,29,34], and two studies reported a few cases subsequently confirmed as schizophrenia [28,45]. Although some studies suggested that psychosis may share genome linkage [51,52] and abnormalities in neurodevelopment [8,9,53], other studies suggested that structural abnormalities differ according to diagnosis and stage [54–56]. In considering the potential variations based on the diagnosis, we analysed the prevalence of CSP in FESZ patients as a separate group (Fig 8; Fig 9).

There were also some limitations caused by sample selection. First, the comparison of the gender effect on CSP was not clear. Three studies [8,27,29] reported no gender difference in CSP prevalence between FEP patients and controls, and two studies [20,29] reported no gender difference in CSP length. On the other hand, one study [34] reported that males had a higher prevalence of a large CSP when all subjects were pooled. However, gender distributions of the included studies were not equally balanced between males and females. For example, a total of 749 FEP patients comprised 477 (63.68%) males and 272 (36.32%) females (Table 1), and the meta-regression by sex ratio (male/female) for the prevalence of “any CSP” suggested heterogeneity in both the FEP patients and healthy controls. Second, the recruitment of FEP patients and healthy controls may have caused a selection bias. Most FEP patients were recruited from hospitals [8,14,16,19,20,27–29,45], except for one study [34] that reported recruitment from a population. Five studies recruited controls from the community [8,14,19,27,34], and other studies [16,20,28,29,45] recruited controls from a mixture of groups, including hospital staff, university students and the community (Table 1).

Although we included longitudinal studies [19,29,34] to explore the increase in the CSP length, the sample sizes were small (FEP patient sample sizes range, 20–75 subjects), and the mean durations of follow-up were short (mean follow-up range, 1.1–2.6 years). The result of our analysis showed no difference in the increase in the CSP length between FEP patients and healthy controls, but one study [34] with a larger sample reported a more significant increase in the CSP length in FEP patients and suggested a main effect of time. Similar to other long-term studies on brain morphometric changes in patients with psychoses [48,57], future studies with longer follow-up times are needed to evaluate the interaction between CSP growth and illness duration.

## Conclusions

Although the combined analysis of the prevalence of “any CSP” showed no statistical significance, the evaluation of study heterogeneity suggested that it is unclear whether “any CSP” is a risk factor for FEP. The current evidence suggests no significant difference in the prevalence of “large CSP” between FEP individuals and healthy controls. Therefore, this evidence is insufficient to support “large CSP” as a risk factor for FEP. The CSP length seems stable during relatively short follow-up times. However, measuring only the CSP length may not be sufficiently sensitive to detect changes in the CSP. Additional longitudinal studies are needed to explore the relationship between morphometric changes in the CSP and the progression of psychosis.

## Appendix: Search strategies

### 1. Medline (Feb 29, 2016)

#1 "septum pellucidum". af.

#2 "septi pellucidi". af.

#3 psychosis [MeSH]

#4 psychotic [MeSH]

#5 schizophrenia [MeSH]

#6 #1 or #2

#7 #3 or #4 or #5

#8 #6 and #7

### 2. Embase (Feb 29, 2016)

#1 "septum pellucidum". af.

#2 "septi pellucidi". af.

#3 exp psychosis/

#4 exp psychotic/

#5 exp schizophrenia/

#6 #1 or #2

#7 #3 or #4 or #5

#8 #6 and #7

### 3. Cochrane Central Register of Controlled Trials (Feb 29, 2016)

#1 "septum pellucidum". af.

#2 "septi pellucidi". af.

#3 "psychosis". af.

#4 psychotic disorders [MeSH]

#5 schizophrenia [MeSH]

#6 #1 or #2

#7 #3 or #4 or #5

#8 #6 and #7

## Supporting information

S1 File MOOSE checklist. (DOC)
