# Supplementary material for: Cavum septum pellucidum and first-episode psychosis: A meta-analysis
Source: PLoS One. 2017 May 17;12(5):e0177715. doi: 10.1371/journal.pone.0177715 (PMC5435239; doi:10.1371/journal.pone.0177715)
Supplement: S1 File — (DOC) [file pone.0177715.s001.doc]

**MOOSE Checklist**

| **Item No** | **Recommendation** | **Brief description** |
| --- | --- | --- |
| Reporting of background should include | | |
| 1 | Problem definition | We investigated the prevalence and changes of cavum septum pellucidum (CSP) in first-episode psychosis (FEP) patients. |
| 2 | Hypothesis statement | Whether the CSP may serve as a risk factor for psychosis. |
| 3 | Description of study outcome(s) | Prevalence and changes of CSP in FEP patients. |
| 4 | Type of exposure or intervention used | First-episode psychosis patients were studied. |
| 5 | Type of study designs used | We included cross-sectional studies and longitudinal studies. |
| 6 | Study population | Patients in included studies were diagnosed with FEP. |
| Reporting of search strategy should include | | |
| 7 | Qualifications of searchers | Two authors, HL and LL were trained in systematic methods of literature searching. |
| 8 | Search strategy, including time period included in the synthesis and key words | Three medical literature databases were searched to identify eligible studies from inception to Feb 29, 2016. Details were in the appendix: search strategies. |
| 9 | Effort to include all available studies, including contact with authors | We contacted the authors and obtained the original study data. Details were in the acknowledgement section. |
| 10 | Databases and registries searched | Medline, Embase, and the Cochrane Central Register of Controlled Trials (CENTRAL) were searched. |
| 11 | Search software used, name and version, including special features used | EndNote was used to merge retrieved citations and eliminate duplications. |
| 12 | Use of hand searching | The bibliography of included studies were examined for further relevant studies |
| 13 | List of citations located and those excluded, including justification | See Fig 1. Article selection flow diagram |
| 14 | Method of addressing articles published in languages other than English | We excluded articles not published in English. |
| 15 | Method of handling abstracts and unpublished studies | We excluded studies not published as full-text. |
| 16 | Description of any contact with authors | We contacted the authors for original study data. |
| Reporting of methods should include | | |
| 17 | Description of relevance or appropriateness of studies assembled for assessing the hypothesis to be tested | All studies provide data on patients with confirmed diagnoses of FEP. |
| 18 | Rationale for the selection and coding of data | Data extracted from studies were diagnoses, sample characteristics, measurement of CSP, outcomes, etc. |
| 19 | Documentation of how data were classified and coded | HL and LL screened eligible titles, abstracts, and full texts independently, evaluated the risk of bias, and collated data from each study. YH and XW inspected the data. |
| 20 | Assessment of confounding | We included only FEP patients and decreased the confounding effects of illness duration and medication use. |
| 21 | Assessment of study quality, including blinding of quality assessors, stratification or regression on possible predictors of study results | Assessment of study quality was conducted by meta-regression and sensitivity analysis. |
| 22 | Assessment of heterogeneity | Assessment of heterogeneity was conducted by subgroup analysis, meta-regression and sensitivity analysis. The heterogeneity of the statistical models was examined via the χ^2^ test and the I^2^ statistic. |
| 23 | Description of statistical methods | Stata version 12.0 was used to analyse the outcome data. Dichotomous data were pooled using the odds ratios (ORs), and the continuous data were pooled using the mean differences (MDs) and associated 95% confidence intervals (CIs). |
| 24 | Provision of appropriate tables and graphics | Forest plots, funnel plots and graphics of univariable meta-regression are drawn. |
| Reporting of results should include | | |
| 25 | Graphic summarizing individual study estimates and overall estimate | Figures 2-10 |
| 26 | Table giving descriptive information for each study included | Tables 1-4 |
| 27 | Results of sensitivity testing | Sensitivity analysis was conducted; effects on heterogeneity were discussed in the paper. |
| 28 | Indication of statistical uncertainty of findings | 95% confidence intervals were given. The heterogeneity analysis of the prevalence of “any CSP” suggested bias in outcome reporting. |
| Reporting of discussion should include | | |
| 29 | Quantitative assessment of bias | Publication bias was assessed using funnel plots, Begg’s and Egger’s tests. |
| 30 | Justification for exclusion | We discussed the results of the sensitivity analyses conducted by leave-one-out method. |
| 31 | Assessment of quality of included studies | We discussed the heterogeneity of MRI measurements, the instability of diagnoses, the selection bias, and durations of follow-up. |
| Reporting of conclusions should include | | |
| 32 | Consideration of alternative explanations for observed results | We consider alternative explanations may be due to the heterogeneity of studies, the measurements of CSP and follow-up times. |
| 33 | Generalization of the conclusions | The results based on current evidence suggest it is unclear whether “any CSP” is a risk factor for FEP due to the heterogeneity of the studies. There is insufficient evidence to support that “large CSP” is a possible risk factor for FEP. |
| 34 | Guidelines for future research | We recommend longitudinal studies to explore the relationship between morphometric changes in the CSP and the progression of psychosis. |
| 35 | Disclosure of funding source | Funding: This project was supported by the Science and Technology Program of Tianjin (No. 15ZXLCSY00020). |
